# Mastication and deglutition changes in children with tonsillar hypertrophy

**DOI:** 10.5935/1808-8694.20130076

**Published:** 2015-10-08

**Authors:** Jaqueline Freitas de Souza, Tais Helena Grechi, Wilma Terezinha Anselmo-Lima, Luciana Vitaliano Voi Trawitzki, Fabiana Cardoso Pereira Valera

**Affiliations:** a Speech and Hearing Therapist (Speech and Hearing Therapist, Department of Ophthalmology, Otorhinolaryngology, and Head and Neck Surgery, School of Medicine of Ribeirão Preto, University of São Paulo).; b PhD (Speech and Hearing Therapist, Department of Ophthalmology, Otorhinolaryngology, and Head and Neck Surgery, School of Medicine of Ribeirão Preto, University of São Paulo).; c Associate Professor (Associate Professor of Otorhinolaryngology, Department of Ophthalmology, Otorhinolaryngology, and Head and Neck Surgery, School of Medicine of Ribeirão Preto, University of São Paulo).; d PhD (Professor of Speech and Hearing Therapy, Department of Ophthalmology, Otorhinolaryngology, and Head and Neck Surgery, School of Medicine of Ribeirão Preto, University of São Paulo).; e Post-doctoral degree holder (Professor, School of Medicine of Ribeirão Preto, University of São Paulo). Faculty of Medicine of Ribeirão Preto - University of São Paulo.

**Keywords:** adenoids, deglutition, mastication, myofunctional therapy, palatine tonsil

## Abstract

The changes in mastication and deglutition in children with adenotonsillar hypertrophy need to be better characterized.

**Objective:**

To evaluate the frequency of parent-reported myofunctional changes and to determine if there are differences in the alteration patterns of children with adenotonsillar hypertrophy and subjects with adenoid hypertrophy.

**Method:**

Questionnaire and assessment by a speech therapist of children aged between three and six years with tonsillar hypertrophy. The data reported by the parents were compared to the data obtained from the speech therapist's evaluation; additionally, data from children with adenotonsillar hypertrophy were compared to findings from subjects with adenoid hypertrophy. Study Design: cross-sectional cohort.

**Results:**

The myofunctional changes observed by the speech therapist were more frequent than the alterations reported by the parents, and there was no correlation between the two findings. The children with adenoid hypertrophy and the individuals with adenotonsillar hypertrophy had the same pattern of myofunctional alteration.

**Conclusion:**

Parents cannot clearly correlate tonsillar hypertrophy with changes in mastication and deglutition. The cause of the respiratory obstruction does not seem to interfere in the pattern of myofunctional change.

## INTRODUCTION

Mouth breathing is observed in subjects with upper airway obstruction. The most commonly involved sites in children are the nose and the pharynx. Allergic rhinitis ranks atop the causes of nasal obstruction (40% to 80% of the cases)^1^, ^2^, followed by deviated septum. Pharyngeal obstructions are also highly prevalent^1^, ^2^, ^3^. Adenoid hypertrophy is the cause of pediatric mouth breathing in 80% of the cases^1^. Adenotonsillar hypertrophy is seen in approximately 46% of pediatric mouth breathers^2^. Orofacial muscle laxity may also lead the mouth to open and give rise to mouth breathing^4^.

Chronic mouth breathing is believed to be associated with various craniofacial and myofunctional alterations, in addition to negatively affecting other functions such as mastication, swallowing, and speech. Craniofacial alterations include maxillary atresia, mandibular retrusion, cross bite, and elongated face in a predominantly dolichofacial pattern^5^, ^6^, ^7^. The most common myofunctional alterations are half-open lips, inferior position of the tongue and tongue laxity^8^, ^9^, choking during mastication^8^, ^9^, ^10^; mastication with half-open lips^8^, ^9^, ^10^; interposition of the lower lip between the incisors; altered swallowing^4^, ^9^, ^11^; inadequate head movements^4^, ^9^; distorted speech, particularly for /n/, /l/4, 9.

Given the associated craniofacial and muscle alterations, parents often report their children have difficulty feeding; according to the parents, their children eat little, slowly, choke frequently, prefer pasty foods, have difficulty chewing and swallowing^8^, ^9^, ^10^, and move their heads to help swallow food, for example^8^. However, the prevalence of deglutition alterations reported by parents of mouth breathers is yet unknown.

Additionally, it is not known whether the deglutition alterations presented by mouth breathing children are due to mechanical obstruction caused by the tonsils or if myofunctional and respiratory alterations could lead to changes in the deglutition pattern. Therefore, this paper features a comparative study involving two groups of individuals, one with subjects with myofunctional and respiratory disorders and without oral obstruction (represented only by children with adenoid hypertrophy) and another with the same alterations in association with mechanical oral obstruction caused by adenotonsillar hypertrophy.

This study aimed to compare the prevalences of parent-reported eating/swallowing complaints and alterations observed in clinical examination by a speech and hearing therapist, and report possible differences between children with adenotonsillar hypertrophy versus children with adenoid hypertrophy.

## METHOD

This study was approved by the hospital's Research Ethics Committee (permit #4346/2008). The parents or guardians of the children included in the study gave informed consent to their participation.

Thirty-four children were enrolled in the study. They were mouth breathers of both genders with deciduous teeth aged between three and six years seen at the Mouth Breathing Ward at University Hospital. The individuals were divided into two groups:

• Ten children with adenoid hypertrophy (Group Ad);
• Twenty-four children with adenotonsillar hypertrophy (Group A+A).

Patients with craniofacial deformities, genetic syndromes, neuromuscular disorders, neurologic alterations, occlusion alterations, individuals previously submitted to orthodontic treatment, ENT surgery, or speech and hearing therapy were excluded.

Enrolled individuals were initially seen by an ENT physician to confirm history of chronic mouth breathing, snoring, and apnea, in addition to relevant ENT symptoms such as signs of allergic rhinitis, recurrent infection, and diurnal symptoms suggestive of apnea in children. The subjects were then submitted to ENT examination, which included anterior rhinoscopy, oral and nasal endoscopy. Adenoid hypertrophy was considered present when nasal endoscopy revealed cavum obstruction by adenoid tissue greater than 70%; adenotonsillar hypertrophy was characterized when individuals had tonsils grade 3 or 4 according to Brodsky^12^. Only children on optimal therapy (topical nasal steroids for at least two months) and without signs of acute disease were included in the study.

Following selection by an otorhinolaryngologist and approval by parents or guardians, a semi-structured questionnaire was answered by the parents or guardians to find whether their children had difficulties feeding, their favorite food consistence, how quickly they ate, and how often the situations listed below were troubling:

• Drinking liquids during meals;
• Eating with the mouth open;
• Food leftovers in the mouth after swallowing;
• Irritability during meals;
• Foods the child refused to eat;
• Respiratory distress while feeding;
• Cough, choking, vomiting, or nausea while feeding.

Patients were then given half a roll of bread and instructed to eat it as they normally would. The individuals sat on a chair while their mastication and deglutition was captured on video with a SONY Hi8 recorder 990x digital zoom, optical 20x CCD-TRV 138 NTSC. The captured footage was assessed at a later stage, considering the following factors:

• Amount of food eaten;
• Teeth preferentially used to cut the food (anterior or posterior teeth);
• Lips open or closed during mastication;
• Interposition of tongue and lips during swallowing;
• Tensioning of the orbicularis oris muscle during deglutition;
• Movements of the head or facial mimicry during deglutition.

Data analysis was performed with the aid of statistical package Graphpad Instat 3.0 for Windows. Fisher's exact test or the Chi-square test were used as needed.

## RESULTS

Thirty-four children were enrolled in this study, 24 (70.6%) on group A+A (adenotonsillar hypertrophy) and 10 (29.4%) on group Ad (adenoid hypertrophy). All children were confirmed to be highly symptomatic mouth breathers, with both snoring and apnea. However, no statistically significant differences between groups were seen for the presence of snoring/apnea (*p* = 1.00). No difference was seen in regards to allergy symptoms (pruritus, sneezing, rhinorrhea), as they were present in 17 (70.8%) patients with adenotonsillar hypertrophy and in five (50%) subjects with adenoid hypertrophy (*p* = 0.2713).

The questionnaire answered by the parents or guardians of the children on symptoms related to feeding and deglutition revealed that, spontaneously and without prior clinical assessment, twelve (50%) children in group A+A and four (40%) in group Ad had difficulty feeding (*p* = 0.7146). However, only 14 (58.3%) patients in the adenotonsillar hypertrophy group and three (30%) in the adenoid hypertrophy group had predominantly solid foods, while 17 children preferred soft, pasty, or liquid foods. No statistically significant differences were seen for preferential food consistence.

Six (25%) parents or guardians of children in group A+A and three (30%) parents or guardians of individuals in group Ad considered their children ate at a normal pace. Slow eating was more commonly reported, with 13 (54.1%) cases in the adenotonsillar hypertrophy group and four (40%) in the adenoid hypertrophy group. The differences between groups were not statistically significant (*p* = 1).

The habit of drinking during meals was reported by 21 (87.5%) parents in the adenotonsillar hypertrophy group and ten (100%) in the adenoid hypertrophy group (*p* = 0.5388). Open lips during mastication occurred systematically in 19 (79.1%) of the children in group A+A and in seven (70%) children with adenoid hypertrophy (*p = 0.6664*). Respiratory distress during feeding was reported for eleven (45.8%) children with adenotonsillar hypertrophy and six (60%) subjects in group Ad (*p* = 0.708); coughing was described for 13 (54.1%) children win group A+A and two (20%) subjects with adenoid hypertrophy (*p* = 0.1413).

Few children had food leftovers in their mouths after swallowing (33.33% of the subjects in group A+A and 10% of the individuals in group Ad) or experienced nausea or vomiting during meals (12.5% of the subjects in group A+A and 20% of the individuals in group Ad).

The results obtained from the examination by a speech and hearing therapist are described on Tables 1 and 2.

**Table 1:** Comparison of frequencies of occurrence of mastication aspects in children with adenotonsillar hypertrophy (group A+A) and children with adenoid hypertrophy (group Ad) based on Fisher's exact test.

Mastication aspects	A+A	Ad	*p*
Bite size			

Large	4 (16.6%)	1 (10%)	
Medium	9 (37.5%)	1 (10%)	0.1735
Small	11 (45.8%)	8 (80%)	

Teeth used to cut food			

Anterior teeth	22 (91.6%)	7 (70%)	0.138
Posterior teeth	2 (8.3%)	3 (30%)	

Lip closure			

Absent	10 (41.6%)	1 (10%)	
Non-systematic	6 (25%)	5 (50%)	0.162
Present	8 (33.3%)	4 (40%)

**Table 2:** Comparison of frequencies of occurrence of deglutition aspects in children with adenotonsillar hypertrophy (group A+A) and children with adenoid hypertrophy (group Ad) based on Fisher's exact test.

Deglutition aspects	A+A	Ad	*p*
Tongue interposition	17 (70.8%)	7 (70%)	1
Lip interposition	6 (25%)	1 (10%)	0.64
Orbicularis oris tensioning	14 (58.3%)	5 (50%)	0.72
Mentalis tensioning	18 (75%)	7 (70%)	1

Proper use of anterior teeth to cut food was seen in 22 (91.6%) patients in group A+A and in seven (70%) subjects in group Ad (*p* = 0.138). Lip closure was systematically present in only eight (33.33%) children in group A+A ad in four (40%) subjects in group Ad (*p* = 0.162). No statistically significant differences were seen in terms of the amount of food swallowed per bite (*p* = 0.1735). The prevalence of myofunctional alterations was similar in both groups.

During deglutition, tongue interposition was seen in 17 (70.8%) patients with adenotonsillar hypertrophy and in seven (70%) individuals with adenoid hypertrophy (*p* = 1). Lip interposition was observed in six (25%) patients in group A+A and in one (10%) individual in group Ad (*p* = 0.6445). No differences were seen between groups for tensioning of the mentalis (18 patients (75%) in group A+A and seven (70%) in group Ad, p = 1) or tensioning of the orbicularis oris muscle (14 children (58.3%) in group A+A and five (50%) in group Ad, *p* = 0.7176). No patients had associated head movements or facial mimicry.

Agreement between the assessments made by the parents or guardians and the speech and hearing therapist was seen in only 20 of the 34 patients. This rate of agreement was deemed low.

## DISCUSSION

Although mastication and deglutition alterations have been well-described for pediatric mouth breathers with adenotonsillar hypertrophy^13^, the relevance of these symptoms for parents or guardians have not been reported in the literature, as nor have the differences between the myofunctional patterns of children with adenotonsillar hypertrophy and subjects with adenoid hypertrophy alone.

All parents and guardians reported their children had respiratory complaints, while only 16 of the 34 respondents alluded to mastication and swallowing disorders, a claim in significant disagreement with the clinical findings observed by the speech and hearing therapist. Such difference of opinion occurred possibly due to the little knowledge parents and guardians have on the repercussions of adenotonsillar hypertrophy and chronic mouth breathing upon the myofunctional system and, consequently, upon mastication and deglutition. On the other hand, knowledge of respiratory repercussions is well established.

In the answers to the questionnaire it was clear that children with hypertrophied tonsils prefer pasty to solid foods, drink more liquids to help mastication, eat with their mouths open more frequently, and choke more often during mastication. These findings are in agreement with another study published by our group^8^.

Most children ate slowly, whether they had adenotonsillar or adenoid hypertrophy. However, this was not the only observed pattern of mastication as reported by other authors^13^. A considerable portion of the children enrolled in this study adopted a faster pace of mastication. This may not be the most common pattern, but it is relatively easy to explain why mouth breathing children may resort to it, as they also use their mouths to breathe^14^. The subjects preferred to take small to medium bites of food, as also described in the literature^13^, ^15^, which can be explained by the concurrent use of the mouth for breathing and eating.

Most children with hypertrophied tonsils could not close their lips while masticating, or did not close them systematically, as described in the literature^8^, ^9^, ^10^, ^11^, ^12^, ^13^. In order to avoid dropping food during deglutition, inadequate lip closure had to be compensated with the interposition of the tongue or lips, or yet by tensioning the orofacial muscles, particularly the mentalis or the buccinator muscles^8^, ^9^, ^10^, ^11^, in an attempt to actively close the lips and allow proper deglutition.

Yet, the authors of this study could not establish a correlation between cause of obstruction and eating patterns. Apparently, respiratory obstruction is associated with significant alterations in mastication and deglutition patterns, regardless the site of origin of the obstruction, while obstruction by the tonsils does not impact myofunctional alterations. No literature was found to support this hypothesis.

Lastly, the data reported in this study indicated that lip closure and projection of the tongue during deglutition were the main alterations found in children with hypertrophied tonsils. Eating alterations were highly prevalent in pediatric mouth breathers and obstruction caused by the tonsils had little impact on the existing myofunctional pattern. Despite the alterations observed during clinical examination, eating-related complaints were not frequently reported by parents or guardians. It is essential to recognize mastication and deglutition alterations that may result in clinical morbidity, along with proper treatments for these children and advice to their parents and guardians.

## CONCLUSION

Parents and guardians reported few deglutition and mastication alterations in children with respiratory obstruction, indicating the need to increase their awareness on the conditions manifested by their children. Respiratory obstruction appears to be more relevant for myofunctional alterations than obstruction per se, whether it is caused by adenoid or adenotonsillar hypertrophy.
